# Biological Activity of Porcine Gastric Mucin on Stress Resistance and Immunomodulation

**DOI:** 10.3390/molecules25132981

**Published:** 2020-06-29

**Authors:** Thiloma D. Liyanage, Pasan S. Dahanayake, Shan L. Edirisinghe, Chamilani Nikapitiya, Gang-Joon Heo, Mahanama De Zoysa, Ilson Whang

**Affiliations:** 1 College of Veterinary Medicine, Chungnam National University, Yuseong-gu, Daejeon 34134, Korea; thilomaliyanage@gmail.com (T.D.L.); shan.lakmal09011@gmail.com (S.L.E.); chamilani14@gmail.com (C.N.); 2 College of Veterinary Medicine, Chungbuk National University, Cheongju 28644, Korea; pasansd@gmail.com (P.S.D.); gjheo@cbu.ac.kr (G.-J.H.); 3 National Marine Biodiversity Institute of Korea (MABIK), 75, Jangsan-ro 101beon-gil, Janghang-eup, Seochun-gun, Chungchungnam-do 33662, Korea

**Keywords:** disease resistance, heat tolerance, immunomodulation, mucin, oxidative stress, zebrafish

## Abstract

Purified porcine gastric mucin (PGM) is an alternative biomaterial to native mucin which displays multifunctional properties for exploring a wide range of biomedical applications. The present study evaluated the in vitro (RAW 264.7 macrophage cells) and in vivo (zebrafish embryos and larvae) bioactivities of PGM. The median lethal concentration (LC_50_) of PGM was 197.9 µg/mL for embryos, while it was non-toxic to RAW 264.7 cells, even at 500 µg/mL. Following PGM exposure (100 µg/mL), a higher embryo hatching rate (59.9%) was observed at 48 h post fertilization, compared to the control (30.6%). Protective effects of PGM from pathogenic *Aeromonas hydrophila* were demonstrated by high larvae survival rates of 85.0% and 94.0% at 50 and 100 μg/mL of PGM exposure, respectively. Heat tolerance effect of PGM (50 and 100 µg/mL) on larvae (40 °C for 48 h) was confirmed by 75% and 100% of survival rates, respectively. Additionally, PGM reduced the *A. hydrophila*–induced reactive oxygen species (ROS) generation in larvae. The qRT-PCR results in PGM exposed larvae exhibited induction of immune-related genes (*tlr5a* and *tlr5b*, *myd88*, *c-rel*, *il1β, tnf-α*, *il6, il10*, *cxcl18b*, *ccl34a.4*, *defbl1*, *hamp*, *ctsd, muc2.1*, *muc5.1*, *muc5.2*, and *muc5.3*), stress response (*hsp70*, *hsp90aa1.1*, and *hsp90ab1*), and antioxidant genes (*cat* and *sod1*). Moreover, our results revealed that PGM involved in the regulation of transcriptional gene induction increases Hsp90 protein in the zebrafish larvae. Furthermore, upregulation of *Il6, Il10, Tnfα, Ccl3, Defa-rs2, Defa21* and *Camp* and antioxidant genes (*Sod2* and *Cat*) were observed in PGM-exposed RAW 264.7 cells. Overall findings confirmed the activation of immune responses, disease resistance against pathogenic bacteria, heat tolerance, and ROS-scavenging properties by PGM, which may provide insights into new applications for PGM as a multifunctional immunomodulator.

## 1. Introduction

Globally, various approaches have been introduced to prevent and control diseases in aquaculture. The use of immunostimulants is a promising and environmentally friendly method to enhance the innate defense mechanisms of fish [1]. In general, immunostimulants consist of biological and synthetic compounds that trigger relevant components of the immune system, to confer protection against specific pathogens. They help to boost the development and strengthen the disease resistance of fish, and these impacts are dependent on the structure and function of different immunostimulants [2].

Mucins are high-molecular-weight glycoproteins produced by a wide range of epithelial tissues in animals. They are composed of a protein backbone that consists of tandem repeats of threonine-and serine-rich amino acid residues, and a large number of *O*-linked carbohydrate side chains [3,4,5,6,7]. Mucins are divided into two groups (secreted and membrane-bound), based on their structure, function, and subcellular localization [8,9,10,11]. The presence of alternating arrays of hydrophilic glycosylated regions and hydrophobic unglycosylated regions ensures the amphiphilic character of mucin [12]. Mucins play a critical role in forming the protective barrier of mucous membranes. In addition, they have several specific functions, including solute transport regulation, establishment of binding sites for commensal and pathogenic microbes, and leukocyte targeting. Moreover, they are known to regulate cellular regeneration, integration, differentiation, cell signaling, cell adhesion, and apoptosis [7,13,14,15].

Native mucins have shown excellent surface adsorption and lubrication properties, which has a potential impact on biomedical applications [16]. Several in vivo infection studies in aquaculture have demonstrated the role of mucins and mucin glycosylation in the defense against fish pathogens [17,18,19]. Similarly, porcine gastric mucin (PGM) has been used as an alternative for native mucin to understand host–pathogen interactions [16,20]. Moreover, PGM has been used in a wide range of supplements, personal hygiene products, and lubricants, as well as in artificial saliva, due to its promising antiviral activity, biocompatibility, and availability [21]. However, studies have also reported cell toxicity effects during cell culture experiments, and inferior effects on virus inhibition upon reconstituted commercial mucin treatments [16,21].

To the best of our knowledge, there has been no study to date that explores the effect of exogenous PGM as a modulator of innate immune responses. The present study focused to understand the immunomodulatory effects of PGM by conducting in vitro assays, using RAW 264.7 macrophage cells and zebrafish (*Danio rerio*), as an appropriate in vivo model. Thereby, we investigated PGM effect on hatching percentage (%), reactive oxygen species (ROS) scavenging properties, disease resistance, thermal (heat) tolerance and immune-modulation upon exposure in zebrafish larvae, and RAW 264.7 cells.

## 2. Results

### 2.1. The In Vivo and In Vitro Toxic Effects of PGM

**Figure 1:**
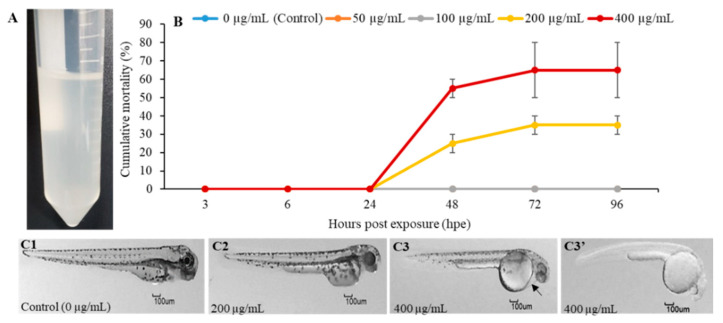
Solubility and effect of purified porcine gastric mucin (PGM) on the zebrafish embryos’ toxicity. (**A**) Type III PGM stock solution (4 mg/mL). (**B**) Mortality of zebrafish embryos exposed to PGM (0–400 μg/mL). Values are means standard error (±SE) of duplicate independent experiments. (**C2–C3′**) Representative examples of malformations caused by PGM exposure (200 and 400 µg/mL) at 96 h post exposure (hpe). (**C1**) Control larva with normal appearance; (**C2**,**C3′**) larva with spinal curvature; (**C3**) (black arrow) a slight pericardial edema in larva; (**C3**,**C3′**) larva with head malformations and underdeveloped embryos.

PGM was completely dissolved in distilled water, without any precipitation, at 4 mg/mL of final concentration. The solution of PGM had a slightly basic pH of 7.1 at 25 °C (Figure 1A). When zebrafish embryos were exposed to PGM (50, 100, 200, and 400 µg/mL), acute mortality was observed only in higher concentrations (>200 µg/mL) of PGM, at 24 h post exposure (hpe). In contrast, PGM-treated embryos at lower concentrations (50 and 100 µg/mL), as well as untreated embryos (control group), had zero mortality throughout the 96 hpe (Figure 1B). The outcome of toxicity results revealed that the zebrafish embryos were least sensitive with median lethal concentration (LC_50_) of 197.9 µg/mL at 96 hpe. Figure 1C2–C3′ depicts the deformities caused by PGM exposure at higher concentrations (200 and 400 µg/mL). Deformities such as spinal curvature (Figure 1C2,C3′), pericardial edema (Figure 1C3), head deformities, and underdeveloped embryos were observed (Figure 1C3,C3′). The typical appearance of larvae without deformities was observed in the untreated control group (Figure 1C1) and PGM-exposed (50 and 100 µg/mL) treatment groups. Thus, we chose exposure doses of 50 and 100 µg/mL as nontoxic concentrations of PGM for in vivo experiments with zebrafish larvae. The cytotoxicity assay (MTT) results revealed that PGM was nontoxic to RAW 264.7 cells up to 500 µg/mL (Figure S1). Hence, we selected 200–400 μg/mL of PGM for in vitro experiments.

### 2.2. Effect of PGM Exposure on Embryo Hatching in Zebrafish

**Figure 2:**
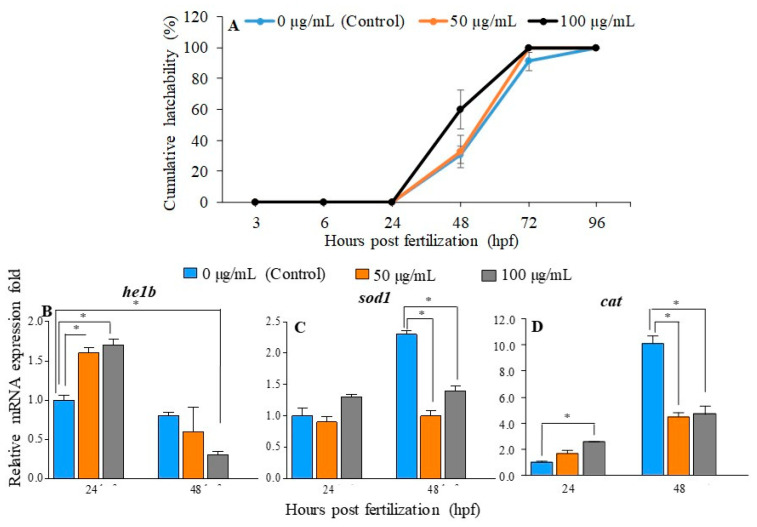
Effect of PGM on the hatching of zebrafish embryos. (**A**) Cumulative hatchability (%) of zebrafish embryos at 50 and 100 µg/mL of PGM exposure. The relative mRNA expression of (**B**) *he-1b*, (**C**) *sod1*, and (**D**) *cat* on hatching upon PGM exposure (50 and 100 µg/mL). Values are mean standard error (±SE) of two independent experiments; asterisk marks are used to indicate the statistical significance compared to untreated control (two-way and one-way ANOVA, * *p* < 0.05).

Figure 2A portrays the effect of PGM exposure on embryo hatching, and we found that there is a non-statistically significant increase in hatchability at 48 h post fertilization (hpf) that disappears by 72 hpf. Following 100 µg/mL PGM exposure, 59.92% hatching was observed at 48 hpf, while 30.56% and 32.98% hatching success was recorded for the control and 50 µg/mL of PGM, respectively. In addition, irrespective of the level of exposure, all embryos were hatched (100%) at 96 hpf. The relationship between early hatching and expression of hatching enzyme 1b (*helb*) was investigated by transcriptional analysis. The expression level of the *helb* gene was significantly higher (*p* < 0.05) in PGM-exposed embryos than in the unexposed control group, at 24 hpf (Figure 2B). The *helb* expression was drastically (*p* < 0.05) reduced up to 0.3-fold at 48 hpf in PGM exposed to a higher dose (100 µg/mL) compared to the control. In addition, the 50 µg/mL of PGM-exposed group showed a downregulation (0.6-fold) which was not significant.

Next, we analyzed the transcriptional expression of *sod1* and *cat* as key antioxidant enzymes, to understand whether early hatching is associated with the level of ROS upon PGM exposure. At 24 hpf, the *sod1* expression was observed at basal levels in the control and PGM-exposed groups, whereas *cat* gene expression was significantly (*p* < 0.05) induced (2.6-fold) in 100 µg/mL PGM-exposed embryos (Figure 2C,D). However, significantly high (*p* < 0.05) *sod1* expression (2.3-fold) was detected in the untreated control group at 48 hpf. In contrast, the *sod1* expression was significantly downregulated (*p* < 0.05) in 50 and 100 µg/mL of PGM exposure (0.9-fold and 1.4-fold, respectively) compared to the control at 48 hpf. The *cat* gene expression was significantly (*p* < 0.05) upregulated (10.1-fold) in the control group at 48 hpf. In addition, PGM-exposed embryos showed significantly (*p* < 0.05) downregulated levels (4.5-fold and 4.7-fold) of *cat* at 50 and 100 µg/mL, respectively, compared to the control at 48 hpf.

### 2.3. Disease Resistance and Heat Tolerance of Zebrafish Larvae upon PGM Exposure

**Figure 3:**
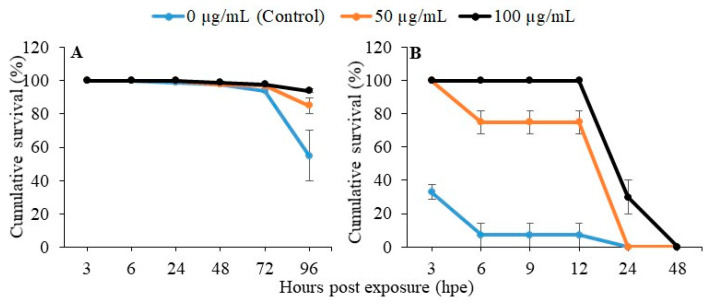
Effect of PGM on disease resistance and thermal tolerance in zebrafish larvae. (**A**) PGM-exposed (50 and 100 µg/mL) zebrafish larvae (at 5 days post exposure (dpe)) were challenged with *A. hydrophila* (2.91 × 10^8^ CFU/mL), and percent survival rate was determined until 96 h post infection (hpi). (**B**) Zebrafish embryos exposed to 50 and 100 µg/mL of PGM and maintained at 28 °C were subjected to heat stress at 40 °C for 48 h, and cumulative survival rate was calculated. The error bars indicate the mean standard error of three replicates.

To determine the disease resistance of PGM exposed larvae, relative percent survival (RPS) was analyzed after *Aeromonas hydrophila* challenge (2.91 × 10^8^ CFU/mL), until 96 h post infection (hpi). No mortality was observed in any of the groups, until 72 hpe. Interestingly, 85% and 94% higher survival rates were displayed in PGM-exposed larvae at 50 and 100 μg/mL, respectively, compared to control (55%) at 96 hpe (Figure 3A).

Thermal tolerance of PGM-exposed larvae was determined by exposure to heat stress at 40 °C for 48 h. Our results clearly indicated the concentration dependent heat tolerance in PGM-exposed (50 and 100 µg/mL) larvae with 75% and 100% survival rate, respectively, compared to the control (7%) at 12 hpe. However, PGM-exposed larvae groups (50 and 100 µg/mL) failed to continue to have a high survival rate and decreased to zero level after 24 and 48 hpe, respectively.

### 2.4. Detoxification Effect of PGM on Bacteria-Induced Oxidative Stress

**Figure 4:**
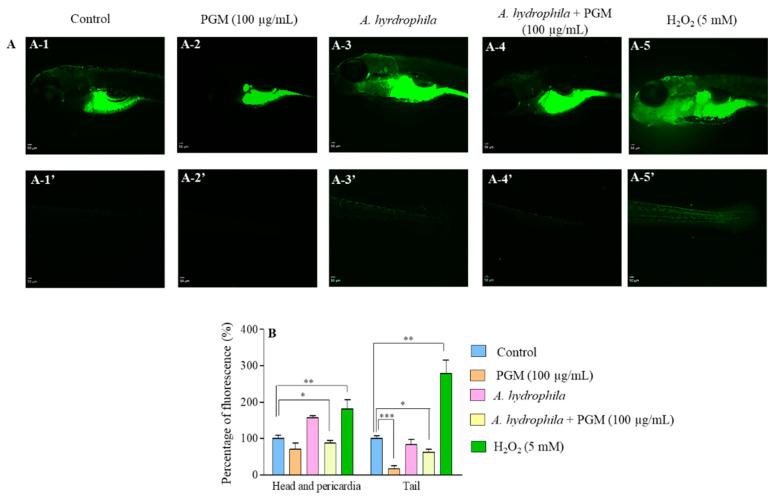
ROS detoxification effect of PGM on *A. hydrophila*–challenged zebrafish larvae. (**A**) Fluorescence images of head, pericardia, and tail areas were detected as follows: control: A-1 and A-1′; PGM (100 µg/mL): A-2 and A-2′; *A. hydrophila* (2.3 × 10^5^ CFU/mL): A-3 and A-3′; *A. hydrophila* + PGM (100 µg/mL): A-4 and A-4′; H_2_O_2_ (5 mM): A-5 and A-5′. (**B**) The graph represents the percentage of fluorescence intensity over the untreated control group. Values were presented as means standard error (±SE), and the asterisk marks are used to indicate the significant difference compared to the respective controls (one-way ANOVA, * *p* < 0.05, ** *p* < 0.01, *** *p* < 0.001).

To examine whether PGM has any effect on reducing the oxidative stress upon *A. hydrophila* exposure, the level of ROS was measured in zebrafish larvae. As expected, the highly visible fluorescent distribution and relative intensity were observed in the head and pericardia (181.2%) and tail (278.4%) of H_2_O_2_ treated larvae (positive control), compared to the control group (100%) without the PGM exposure (Figure 4A,B). The second highest fluorescent level was shown in the head and pericardia (156.5%) of *A. hydrophila*–infected larvae, indicating that bacteria challenge causes the accumulation of ROS. As expected, the relative fluorescence was decreased in the group of *A. hydrophila*–infected larvae with the PGM treatment (head and pericardia: 87.4%, and tail: 62.3%), compared to *A. hydrophila*–infected larvae; however, the ROS-scavenging effect was not significant. Furthermore, the results evidenced that significantly (*p* < 0.05) decreased the fluorescence level in the PGM-exposed *A. hydrophila*–infected larvae, as compared to the control group. The lowest level of fluorescence (16.6%) was in the tail of the larvae group exposed only to PGM, compared to the untreated control group. The PGM-treatment groups showed a similar fluorescence pattern in the head, pericardia, and tail, except the *A. hydrophila* treatment group. The overall results revealed PGM’s ability to decrease the oxidative stress upon *A. hydrophila* exposure.

### 2.5. Transcriptional Analysis of Immune-Related Genes in PGM-Exposed Zebrafish Larvae

**Figure 5:**
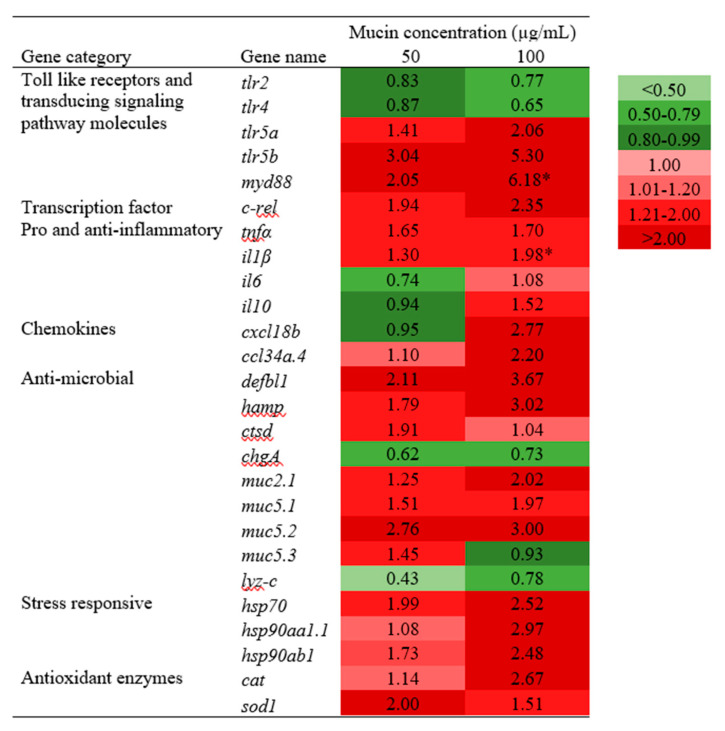
The relative mRNA expression of immune and antioxidant genes in PGM-exposed zebrafish larvae. Embryos (2 days post fertilization (dpf)) were exposed to PGM (0, 50, and 100 µg/mL), and larvae were sampled at 7 dpe for isolating RNA. The asterisk marks are used to indicate the statistical significance compared to the control. Basal expression level was considered as 1.00-fold; upregulated and downregulated expressions were considered as >1.01-fold and <0.80-fold, respectively (one-way ANOVA, * *p* < 0.05).

Figure 5 interprets the relative mRNA expression levels of immune and antioxidant genes in PGM-exposed larvae for seven days. PGM-exposure (50 and 100 μg/mL) demonstrated distinct immune and antioxidant gene expression profiles in the larvae, compared to the control group. Among the selected TLRs, *tlr5a* and *tlr5b* showed concentration-dependent upregulation (>2-fold). Moreover, the transducing signaling pathway molecule (*myd88*) increased the expression (6.18-fold). A similar upregulation pattern was observed for transcription factor *c-rel* gene at 50 μg/mL (1.94-fold) and 100 μg/mL (2.35-fold) of PGM exposure (1.94-fold and 2.35-fold). Considering pro- and anti-inflammatory genes, *tnf-α* and *il1β* showed concentration-dependent upregulation (>1.3-fold), where the expression was significant for *il1β* (1.98-fold) at 100 μg/mL. In contrast, a 1.52-fold induction was observed at 100 μg/mL for *il10*, whereas no upregulation was observed for *il6*. In the group of chemokines, both *cxcl18b* and *ccl34a.4* were induced by >2-folds at the highest concentration (100 μg/mL). Among the antimicrobial genes, *defbl*, *hamp*, *muc2.1*, *muc5.1*, and *muc5.2* showed concentration-dependent upregulations (>1.2-fold). In contrast, *ctsd* and *muc5.3* were upregulated only at 50 μg/mL (1.91-fold), while *chgA* and *lyz-c* showed no upregulation upon exposure. The stress-responsive genes *hsp70* (1.99-fold and 2.52-fold), *hsp90aa1.1* (1.08-fold and 2.97-fold), and *hsp90ab1* (1.73-fold and 2.48-fold) resulted in concentration-dependent upregulation at 50 and 100 μg/mL, respectively. Additionally, the antioxidant enzyme *cat* showed slight upregulation (>1.14-fold), whereas *sod1* was upregulated over 2.00-fold at 50 μg/mL.

### 2.6. PGM Effect on Hsp90 Protein Expression

**Figure 6:**
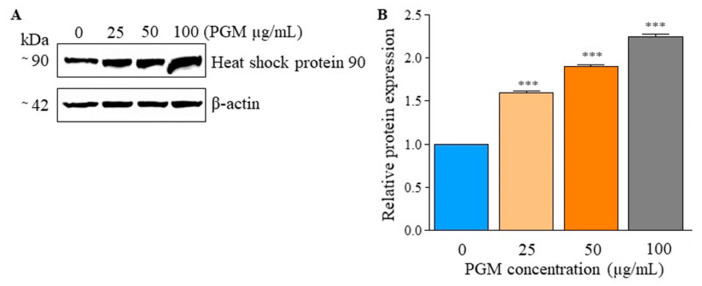
Expression of Hsp90 in PGM-exposed zebrafish larvae by immunoblot analysis. (**A**) Expression levels of Hsp90 in PGM-exposed (0, 25, 50, and 100 µg/mL) larvae are shown (**B**) Quantitative analysis of Hsp90 expression in larvae is displayed with β-actin expression, relative to the untreated control. The asterisk marks are used to indicate statistical significance compared to the control (one-way ANOVA, *** *p* < 0.001).

According to Figure 6A, PGM exposure (25, 50, and 100 μg/mL) resulted in a concentration-dependent increase in Hsp90 protein in zebrafish larvae. A similar expression pattern was observed with the quantitative analysis, in which the relative Hsp90 protein expression was highest at 100 μg/mL of PGM exposure (2.25-fold), as compared to the control (Figure 6B). Among all the treatments, almost-equal expression levels of β-actin were observed.

### 2.7. Transcriptional Analysis of Immune-Related Genes in PGM-Exposed RAW 264.7 Cells

**Figure 7:**
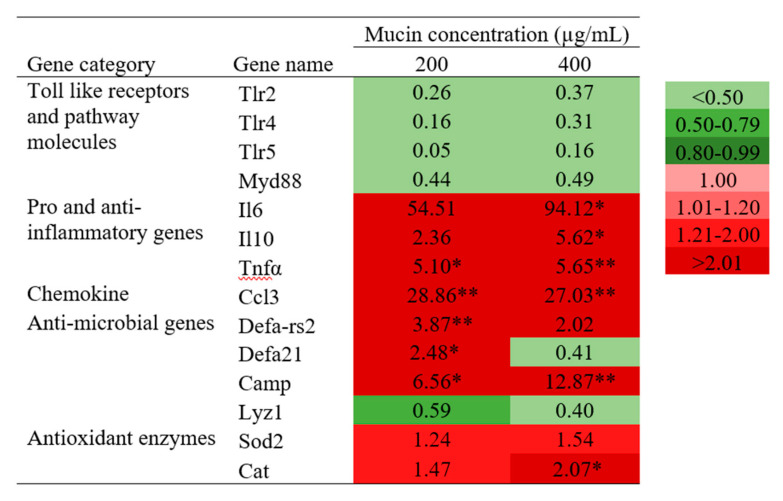
The relative mRNA expression of immune and antioxidant genes in PGM-treated RAW 264.7 cells. Cells were treated with PGM (200 and 400 µg/mL) for 24 h. The asterisk marks are used to indicate statistical significance compared with the control. Basal expression level was considered as 1.00-fold; upregulated and downregulated expressions were considered as >1.01 and <0.80-folds, respectively (one-way ANOVA, * *p* < 0.05, ** *p* < 0.01).

The mRNA expression levels of immune and antioxidant genes in RAW 264.7 are shown in Figure 7. The upregulation of pro-inflammatory genes (*Il6* and *Tnf-α*), anti-inflammatory (*Il10*), chemokine (*Ccl3*), antimicrobial genes (*Defa-rs2*, *Defa21*, and *Camp*), and antioxidant genes (*Sod2* and *Cat*) was observed, while Toll-like receptors and signaling pathway molecules were downregulated. Particularly significant expression was detected for *Tnf-α* (5.10-fold and 5.65-fold), *Ccl3* (28.86-fold and 27.03-fold), and *Camp* (6.56-fold and 12.87-fold) genes at 200 and 400 µg/mL of PGM exposure, respectively. Furthermore, significantly upregulated expression was shown for *Il6*, *Il10*, and *Cat* genes in PGM-treated RAW 264.7 cells at 400 µg/mL.

## 3. Discussion

In this study, we evaluated the toxicity effect of PGM on zebrafish embryos at different PGM concentrations, since performing toxicity and safety assessments is essential during the development of a new candidate of immunostimulants. Any compound can be toxic at higher doses; mostly, they are safe at very low levels. Moreover, the use of additives in the purification process of commercial mucin may be associated with the toxicity effects [16]. The signs of deformities were seen at 96 hpe when the cumulative mortality rate was recorded as >35% and >65% for the 200 and 400 µg/mL concentrations of PGM, respectively. Therefore, as denoted by the LC_50_, we suggest that the safe concentration for the zebrafish would be ≤197.9 µg/mL of PGM. Moreover, Lieleg et al. (2012) [21] have reported that commercial PGM was not toxic to HeLa cells at 1% (*w*/*v*) solution at pH 7. Similarly, when RAW 264.7 cells were exposed to PGM, it was not toxic up to 500 µg/mL. Collectively, these in vitro and in vivo nontoxic concentrations were considered to be biologically safe doses for our further studies.

Several factors, including environmental, physiological, biological, and genetic characteristics, may influence the fish-hatching process [22,23]. For instance, the *he1a* and *he1b* genes are induced prior to the hatching of zebrafish larvae [23]. Although the induced *he1b* expression was observed for both 50 and 100 µg/mL of PGM at 24 hpf, a lower hatchability % was noted for lower doses of PGM (50 µg/mL) at 48 hpf. This difference might be due to the lack of one or more aforementioned factors.

Antioxidant enzymes such as SOD and CAT play an important role in mitigating and repairing the harmful effects of ROS by converting superoxide anion O_2_^−^ into H_2_O_2_, and finally degrading into H_2_O. Therefore, antioxidant enzymes assist in ensuring the antioxidant protection against deleterious ROS effects [24]. ROS are formed as a natural by-product during the normal metabolism of oxygen and are important in regular cell functioning [25]. Markedly, the increased expression of *sod1* and *cat* in the control at 48 hpf may have resulted from increased metabolic demand during hatching. However, *sod1* and *cat* expressions were reduced when compared to that of the control at 48 hpf. PGM consist of ROS-scavenging properties in non-mucin components which enhance mucin’s capacity to quench ROS [26]. In this study, a significantly lower expression of *sod1* and *cat* upon PGM exposure confirmed its extensive ROS-scavenging properties. Therefore, this involvement may have caused a significant increase in the number of successfully hatched embryos.

Immunomodulation has the potential to elevate the larval survival rate by enhancing their innate immune responses until adaptive immunity becomes fully effective [27]. Previous studies have demonstrated the prophylactic use of immunostimulants to enhance the innate immune system of zebrafish against pathogenic infections during various stress conditions [28,29,30,31]. Interestingly, our study revealed the importance of PGM exposure on zebrafish larvae against *A. hydrophila* infection. Furthermore, results suggest that 100 μg/mL PGM is the most effective concentration for zebrafish larvae to activate innate immune responses against *A. hydrophila* infection. This clarifies the critical role of mucin as an exogenous stimulant in host-cell microbial interaction for the first time. The experiment result obtained from the thermal-tolerance activity of PGM revealed significant heat-stable properties of PGM, minimizing heat stress during zebrafish larval development. The PGM exposure (100 μg/mL) emphasizes its higher thermal tolerance capacity, with 100% survival rate and dose-dependent effectiveness at early stage of heat stress. This might be attributed to the functional role of membrane-associated MUC1-C in attenuating the activation of intrinsic apoptotic pathway and its interaction with heat-shock proteins (HSP70–HSP90) [32]. However, none of the concentrations was able to support larvae that were tolerant to the heat stress at 48 hpe.

In fish, ROSs are scavenged by non-enzymatic antioxidants and antioxidant enzymes [33]. Hong et al. (2015) [34] have suggested that the presence of mucins that are rich in threonine enhance the functions of fish digestive system by decreasing ROS level in common carp (*Cyprinuscarpio* L.). Previous in vitro biochemical assays also reported the anti-hydroxyl radical (AHR) scavenging capacity of PGM [35]. In this study, PGM significantly suppressed the ROS levels induced by *A. hydrophila* exposure in zebrafish larvae. Furthermore, it was confirmed that the presence of PGM alone reduces the ROS levels in the zebrafish larvae. These results suggest the potential role of PGM as an effective ROS-scavenging activity to alleviate bacteria-induced oxidative stress, and thus protecting cells from apoptosis.

The soluble immunostimulants are usually absorbed by mucosal tissues and may subsequently activate immune responses in fish. This strategy has been suggested in protecting larval fish, as soluble immunostimulants can be easily taken up through their mucosal-epithelial barriers existing in organs such as gills and skin [36,37,38]. Therefore, PGM is an ideal immunostimulant for activating immune cells such as macrophages and lymphocytes and initiate further defense mechanisms. Toll-like receptors (TLRs) play a vital role in innate immunity by triggering the first line of defense against invasive pathogens [39]. Particularly, the TLR5 is known as the only protein-binding TLR that is conserved in vertebrates [40,41,42]. Hence, we could postulate one of the main roles of mucin as transcriptional activation of *tlr5a* and *tlr5b*. Following TLR5 stimulation and induction of *myd88*-dependent proinflammatory transcription factor, NF-κB, in various cells, such as epithelial cells, monocytes, and dendritic cells, may result in the activation of downstream innate immune responses [43]. The MYD88 is a critical adaptor protein in the TLR signaling pathway, as it is used by all TLRs except for TLR3 [44]. Van der Vaart et al. (2013) [45] demonstrated the protective role of MyD88 during early mycobacterial pathogenesis in zebrafish larvae. As a member of the transcription factor family NF-κB proteins, c-Rel modulates genes regulating multiple cellular processes from apoptosis to proliferation and inflammation [46]. Inflammatory cytokines such as IL-1β and TNF-α can activate the signaling pathway of the inflammatory responses [47]. The *il1β* is one of the early responding pro-inflammatory cytokines that allows organisms to react against infectious insults, stimulating an inflammatory cascade, along with other defensive reactions [48]. Significant upregulation of *il1β* suggests the involvement of mucin in activating the signaling pathway of inflammatory responses. Chemokines (*cxcl18b* and *ccl34a.4*) are ubiquitous cytokine molecules that also play a significant role in the immunological and physiological functions of the fish [49].

Cobo et al. (2015) [50] suggested the involvement of MUC2 in regulating the expression of the antimicrobial activity of β-defensin 2, where in vivo mice experiments exhibited reduced expression of β-defensin in Muc2^−/−^ (homozygous) in mice colon, when compared to mice with Muc2^+/−^ (heterozygous) and Muc2^+/+^. Similarly, in this study, upon PGM exposure, the upregulation of endogenous *muc2.1* and other mucin genes may have induced the expression of antimicrobial peptides such as β defensin1, hepcidin, and cathepsin *D.* Concentration-dependent PGM exposure upregulated *hsp70*, *hsp90aa1.1*, and *hsp90ab1* mRNA levels at 7 days post exposure (dpe), and it is further evidenced by the induction of Hsp90 by Western blotting. This also may have resulted due to the functional role of MUC1-C, as we described earlier for heat resistance of zebrafish larvae at 5 dpe [32]. In addition, Hsp70 and Hsp90 may be responsible for targeting MUC-1C to the mitochondrial outer membrane through the delivery of proteins [32]. These proteins actively protect and recover the functions of various protein complexes [51]. Importantly, Hsp90 is noncovalently associated with the immunogenic peptides, highlighting their potential role in regulating immune responses [52]. Therefore, a high abundance of Hsp90 protein suggests that mucin may alter the immune responses in zebrafish. The upregulation of antioxidant enzymes (*cat* and *sod1*) further confirms that mucin may reduce oxidative stress by triggering the release of antioxidant-related genes, to overcome the stress conditions. Overall results suggest the active role of PGM as an immunostimulant in regulating immune responses.

Kono and Sakai (2001) [53] reported the activation of macrophages and lymphocytes in fish after being treated with immunostimulants. Antigen-presenting cells such as macrophages express pathogen recognition receptors (PRRs), which are used for the detection of pathogen-associated molecular patterns (PAMPs) [54]. Macrophages play a key role in inflammatory responses by secreting pro- and anti-inflammatory cytokines such as IL-1β, IL-6, TNF-α, and IL-10 [55]. Therefore, the significant expression of pro-inflammatory genes in PGM-exposed RAW 264.7 cells reveals that it may activate the immune response pathways similar to zebrafish in this study. This was further confirmed by the induced expression of antimicrobial and antioxidant genes which were observed similar to zebrafish. The reason for downregulated Toll-like receptors and pathway molecules perhaps related to the upregulation of an anti-inflammatory cytokine (Il-10). IL-10 is involved in inhibiting pro-inflammatory cytokines, expression of anti-inflammatory responses, and phagocytic activity [56]. Therefore, the induced pro- and anti-inflammatory cytokines by PGM exposure highlight its immune modulatory role in RAW cells.

## 4. Materials and Methods

### 4.1. Preparation of PGM Solution

To prepare a stock solution, 200 mg of type III PGM (Sigma-Aldrich, St Louis, MO, USA) was dissolved in 50 mL autoclaved distilled water, at room temperature (25 °C). The pH of the stock solution of PGM (4 mg/mL) was measured by using a digital pH meter (Thermo Scientific, Waltham, MA, USA). The stock solution was diluted with autoclaved distilled water, according to the required concentration of PGM for different assays.

### 4.2. Cell Culture and Cytotoxicity of PGM

The RAW 264.7 murine macrophage cells were cultured in Dulbecco’s modified Eagle medium (WELGENE Inc., Daegu, Korea) with 10% (*v*/*v*) fetal bovine serum (WELGENE Inc., Daegu, Korea) and antibiotic–antimycotic solution (Gibco™, GrandIsland, NY, USA). The cells were incubated at 37 °C, in a humidified atmosphere containing 5% CO_2_, and the medium was replaced daily. Cells were seeded (100 µL/well) in a 96-well flat-bottom microtiter plate, at a density of 2.0 × 10^5^ cells/mL, and allowed to adhere overnight. The medium was replaced, and cell cytotoxicity was determined by MTT (3-[4,5-dimethylthiazol-2-yl]-2,5-diphenyltetrazolium bromide; Sigma-Aldrich, St Louis, MO, USA) assay. In brief, cells were treated for 24 h with 10–500 µg/mL of PGM. Autoclaved distilled water was used as the negative control. After incubation, the medium was replaced, and 10 μL of MTT solution (5 mg/mL in phosphate-buffered saline (PBS)) was added to each well and incubated for 4 h at 37 °C. The medium was removed, and 50 μL of dimethyl sulfoxide (DMSO; Sigma-Aldrich, St Louis, MO, USA) was added, to solubilize the formazan crystals. The absorbance was measured at 595 nm, using a microplate spectrophotometer (Bio-Rad Laboratories, Inc., Richmond, CA, USA).

### 4.3. Determination of In Vivo Toxicity of PGM on Zebrafish Embryos

Wild-type adult zebrafish were raised at 28.5 °C, in an automated circulatory system, under a 14:10 h light:dark schedule. Embryos were obtained and raised until larvae stage (7 dpf), in standard Petri dishes (90 × 20 mm) containing embryo water with aquarium salt (60 mg/L), at 28.5 °C. Healthy zebrafish embryos (2 hpf) were selected, and 10 embryos per well (3 replicates/treatment) were incubated in 6-well plates with 10 mL of embryonic water. Then the embryos were exposed to serially diluted PGM (50, 100, 200, and 400 μg/mL). The embryonic water was used as the control. Mortality was observed up to 96 hpe. To determine the nontoxic levels of PGM to embryos, LC_50_ was calculated (https://www.aatbio.com/tools/lc50-calculator). Additionally, phenotypic abnormalities in zebrafish larvae upon PGM exposure were observed, using light microscopy (Nikon, SMZ1000, Tokyo, Japan), and the images were captured with a digital camera (Moticam Pro205, San Antonio, TX, USA), at 96 hpe. The experiment was carried out twice, to increase the accuracy. After determining the nontoxic concentrations, 50 and 100 μg/mL of PGM were selected for further experiments.

### 4.4. Effect of PGM on Zebrafish Hatching

To examine the effect of PGM on hatching, zebrafish embryos were exposed to 50 and 100 μg/mL of PGM, as mentioned in Section 4.3. Then the hatchability was monitored, and the hatching rate was calculated up to 48 hpe. In addition, embryos (n = 50) were placed in Petri dishes containing 50 mL of embryonic water (control) and similar concentrations of PGM. The exposed embryos and hatched larvae were collected at 24 and 48 hpe, snap-frozen in liquid nitrogen, and then stored at −80 °C, until RNA isolation. To increase the accuracy, the experiment was carried out twice.

### 4.5. PGM Effect on A. hydrophila Challenge and Heat Resistance in Zebrafish Larvae

To investigate the disease-resistance capacity of PGM, zebrafish embryos were exposed to 50 and 100 μg/mL of PGM, as mentioned in Section 4.3. The PGM-exposed larvae and the control larvae (without PGM exposure) at 5 dpf were bath-immersed with pathogenic *A. hydrophila* (2.91 × 10^8^ CFU/mL), and the cumulative survivals were recorded until 96 hpi. To examine the heat resistance of PGM on zebrafish larvae, PGM-exposed embryos were maintained at 28 °C, and 5 dpf larvae were subjected to heat stress by incubating at 40 °C. The cumulative survivals were recorded until 48 hpe, and the experiment was carried out twice.

### 4.6. Analysis of ROS Production in PGM Exposed Larvae Upon A. hydrophila Challenge

To measure the effect of PGM on ROS detoxification, zebrafish larvae were grouped as (1) control, (2) PGM (100 μg/mL), (3) *A. hydrophila* (2.3 × 10^5^ CFU/mL), (4) PGM (100 μg/mL) + *A. hydrophila*, and (5) positive control, H_2_O_2_ (5 mM). The embryos in Groups 2 and 3 were exposed to 100 μg/mL of PGM, as described under Section 4.3. After 5 dpf, the larvae in Groups 3 and 4 were challenged with *A. hydrophila*, according to the method described in Section 4.5. The level of ROS in larvae in all the groups was measured at 48 hpi. Initially, larvae were stained, using (5 μg/mL) fluorescent dye, 2′7′dichloro-dihydro-fluorescein diacetate (H_2_DCFDA; Sigma-Aldrich, St Louis, MO, USA), for 30 min, at 28 °C, in the dark. The excess dye was washed 4 times, and intracellular ROS generation in the head, pericardia, and tail areas was detected under a microscope (Nikon SMZ1000, Tokyo, Japan) equipped with fluorescence filter (Chroma, Bellows Falls, VT, USA). The fluorescence intensity of individual larvae was quantified by ImageJ software (ImageJ, version 1.6, Bethesda, MD, USA), and the percentage of fluorescence intensity was calculated compared to the untreated control.

### 4.7. Isolation of Total RNA and Quantitative Real-Time Polymerase Chain Reaction (qRT-PCR)

To determine the immune modulation effect of PGM, qRT-PCR) was performed in larvae exposed to PGM from the embryonic stage and in PGM-treated RAW 264.7 cells. Briefly, RAW 264.7 cells (2.0 × 10^5^ cells/mL) were seeded in a 6-well plate and allowed to adhere overnight (3 replicates/treatment). Cells were maintained as described in the Section 4.3. After replacing the medium, the cells were exposed to 200 and 400 μg/mL of PGM, at 37 °C, for 24 h, in a 5% CO_2_ incubator_._ Following the aspiration of the medium, cells were washed in PBS and centrifuged at 300 g for 5 min. After complete removal of the supernatant, the cell pellet was stored at −80 °C, until isolation of RNA. In addition, 50 embryos containing 50 mL of embryonic water in Petri dishes were exposed to 50 and 100 μg/mL of PGM, until 7 dpe, and the larvae were snap-frozen in liquid nitrogen and stored at −80 °C, until RNA isolation. Total RNA was extracted by using Tri Reagent^TM^ kit (Sigma-Aldrich, St Louis, MO, USA), following the manufacturer’s protocol. A total of 2.5 µg of total RNA was reverse-transcribed by the Prime Script™ first-strand cDNA synthesis kit (TaKaRa^®^, Tokyo, Japan), diluted 40 ×, and stored at −20 °C. The qRT-PCR was performed to analyze the gene expression in PGM-treated cells, larvae, and controls, using a Thermal Cycler Dice Real Time System (TaKaRa, Tokyo, Japan). Zebrafish *β-actin* and mouse Gapdh were used as the housekeeping genes, and the reaction was performed according to a method described in our previous study [57]. The expression fold was calculated by the 2^−(∆∆CT)^ method [58]. The description of the gene-specific primer sequences is shown in Supplementary Table S1.

### 4.8. Immunoblot Analysis for Hsp90 Expression in Zebrafish Larvae

For analyzing the Hsp90 protein expression, zebrafish embryos (n = 35) were exposed to 25, 50, and 100 μg/mL of PGM, until 7 dpe. Then, the collected larvae were snap-frozen in liquid nitrogen and stored at −80 °C, till use. The larvae were homogenized in a lysis buffer, pH 7.6 (ProEX^TM^ CETi, TransLab Inc, Daejeon, Korea) and protein concentration was measured by using Bradford assay (Bio-Rad Laboratories, Inc., Hercules, CA, USA). Samples were then denatured with 2x Laemmli sample buffer (Sigma Aldrich, St Louis, MO, USA), at 100 °C, for 5 min. A sample (50 µg) of each PGM-treated and control group was loaded and separated by 10% SDS-PAGE, followed by transferring onto an Immobilon-P polyvinylidene difluoride (PVDF) membrane (Millipore, Bedford, MA, USA) for 2 h, using Trans-Blot semidry transfer cell (Bio-Rad Laboratories, Inc., Hercules, CA, USA). The membrane was blocked for 1 h in Tris-buffered saline with 5% bovine serum albumin (BSA) and 0.05% Tween20 (TBST), followed by overnight incubation with primary antibodies, anti-heat-shock protein 90-Hsp90 (Cell Signaling Technology; #4874) and anti-β-actin (C4) (Santa Cruz Biotechnology; SC-47778) at 4 °C. On the following day, membranes were washed 3 times with PBS containing 0.05% Tween20 (PBST), for 10 min, and incubated at room temperature with 1:3000 of horseradish peroxidase (HRP)-conjugated secondary antibodies, anti-rabbit IgG antibody (HRP) (Cell Signaling Technology; #7074) for Hsp90, and mouse IgG antibody (HRP) (GeneTex; GTX213111-01) for β-actin in TBST, for 1 h, respectively. Then, the membranes were washed 3 times, for 10 min, with PBST, followed by HRP developing with Western blotting detection reagent (Western Femto ECL Kit, LPS Solution, Daejeon, Korea). This was visualized by using a chemiluminescence detection system (Fusion Solo S, Vilber, Lourmat, France). Simultaneously, protein bands of Hsp90 and β-actin were quantified by Evolution-CAPT software (FUSION software user and service manual—v17.03, Vilber, Lourmat, France) and normalized with the β-actin, to find the relative Hsp90 protein expression compared to the control.

### 4.9. Statistical Analysis

The statistical differences of cumulative mortality, hatchability, survival upon *A. hydrophila* challenge, and heat stress were analyzed at each time point, compared to that of the control, using one-way analysis of variance (ANOVA). To assess the concentration- and time-dependent *he1b* mRNA expression analysis, two-way ANOVA was used. The significant differences of *sod1* and *cat* mRNA expressions were analyzed, comparing with the controls at respective time point, using one-way ANOVA. The statistical significance of fluorescence percentage and other experiments on mRNA expressions were analyzed, compared to that of the control, using one-way ANOVA. Tukey’s test was used for the mean comparison. The significant differences were defined at *p* < 0.05, and data were presented as mean ± SE for three replicates. The data were statistically analyzed, using the GraphPad Prism software version 5 (GraphPad Software Inc., San Diego, CA, USA).

## 5. Conclusions

Collectively, our results suggest that PGM may potentially be involved with the embryonic hatching process and ROS-scavenging activity, as well as enhance the disease and thermal resistance, and immunomodulation in zebrafish larvae. This study revealed that PGM is involved in the regulation of transcriptional gene induction to increase Hsp90 protein in the zebrafish larvae. Particularly, this study is the first to use exogenous PGM as an immunostimulant in fish. Hence, our overall findings shed light on the potential applications of PGM as a biomaterial which modulates the immune system of fish and thereby protects the host from pathogenic invasions. Furthermore, we believe that this study may ultimately contribute to develop PGM-based therapeutic strategies to control and prevent diseases in fish.

## Supplementary Materials

The following are available online. Figure S1. Effect of PGM on the viability of RAW 264.7 cells. The cells were treated with PGM (0–500 µg/mL) for 24 h, and cell viability was assessed by MTT assay. The results were expressed as the percentage of surviving cells over untreated control cells. Values were presented as means ± SE. Table S1. Description of the selected gene specific primers used in this study.
