# Supplementary material for: Biological Activity of Porcine Gastric Mucin on Stress Resistance and Immunomodulation
Source: Molecules. 2020 Jun 29;25(13):2981. doi: 10.3390/molecules25132981 (PMC7411864; doi:10.3390/molecules25132981)
Supplement: Supplementary file 1 [file molecules-25-02981-s001.pdf]

SUPPLEMENTARY MATERIALS

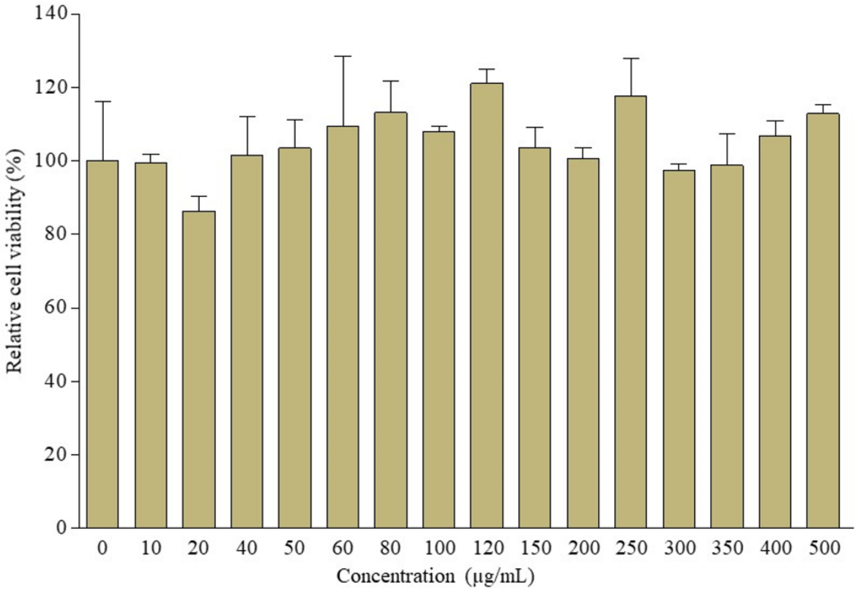

Figure 1. Effect of PGM on the viability of RAW 264.7 cells. The cells were treated with PGM (0-500 μg/mL) for 24 h and cell viability was assessed by MTT assay. The results were expressed as the percentage of surviving cells over untreated control cells. Values were presented as means ± SE.

Supplementary table 1. Description of the selected gene specific primers used in this study.

Gene	Accession number	Primer name	Primer sequence (5'-3')
		**Zebrafish genes**	
Hatching enzyme 1b (he1b)	NM_213635.2	he1b-F	GCTTTGTGCCCAGATCAATTC
		he1b-R	CCTGTTTGCCACCAGTTCTA
Toll like receptor 2 (tlr2)	NM_212812.1	tlr-2-F	TCTCCGTCTTGGTTTCAC
		tlr-2-R	GGTCCCACAGTTGAGTATG
Toll like receptor 4 (tlr4)	AY388400.1	tlr-4-F	GGAATAATGGGCAGCCGTAAG
		tlr-4-R	AGCGACACCAACTATCAATG
Toll like receptor 5 (tlr5a)	AY389449.1	tlr-5-F	ACTCCGCTGTTGCTTTGA
		tlr-5-R	GTTTAGACACCACGCAAATGG
Toll like receptor 5 (tlr5b)	BC163185.1	tlr-5-F	GAAACATTCACCCTGGCACA
		tlr-5-R	CTACAACCAGCACCACCAGAATG
Myeloid differentiation primary response factor 88 (myd88)	DQ100359.1	myd88-F	AACAACTTCGCTGGATAA
		myd88-R	GTTACTGGAATCGCCTCA
c-rel	AY163837	c-rel-F	ACTACAGCTCCCAACAGCCTCAAA
		c-rel-R	AAACTGGTAGCCCGTTGCTAGTGA
Tumor necrosis factor- α (tnfα)	AF025305	tnf- α-F	AGAAGGAGAGTTGCCTTTACCGCT
		tnf- α-R	AACACCCTCCATACACCCGACTTT
Interleukin-1β (il1β)	AY340959.1	il-1β-F	TCAAACCCCAATCCACAGAG
		il-1β-R	TCACTTCACGCTCTTGGATG
Interleukin-6 (il6)	JN698962.1	il-6-F	TCAACTTCTCCAGCGTGATG

		il-6-R	TCTTTCCCTCTTTTCCTCCTG
Interleukin-10 (il10)	AY887900.1	il-10-F	CCCTATGGATGTCACGTCATG
		il-10-R	CATATCCCGCTTGAGTTCCTG
Chemokine ligand 18b (cxcl18b)	NM_001115060	cxcl-18b-F	CTGCTGCTGCCGGTAGTTTA
		cxcl-18b-R	TCAACTTTGTCGCAGTTTGG
CC-chemokine (ccl34 a.4)	BC162421.1	ccl34 a.4-F	TGCAGCTCAACCAGAAGATG
		ccl34 a.4-R	CTTTGACGCATGGAGGATTT
Defensin, beta-like 1 (defbl1)	NM_001081553.1	defbl1-F	CTGCTTGTCCTTGTCGTACT
		defbl1-F	GCAAACACACTCCTTGTCTG
Hepcidin, transcript variant 1 (hamp)	NM_205583.2	hamp-F	CATACAGCAGGTACAGGATGAG
		hamp-R	GAGGGTCTGTTAGTCTGTGTTC
Cathepsin D (ctsd)	NM_131710.2	ctsd-F	GAACCCTCAGACGAACACTAAG
		ctsd-R	GGGAAGCCCAGGTTGTATTT
Chromogranin A (chga)	NM_001006059.2	chga-F	GAGGAAGACCACAGTAAGGAAC
		chga-R	CTGTCTTCGGCTCTGGATTT
Mucin 2.1 (muc2.1)	ENSDARG00000074142	muc2.1-F	AATATGCCTTGCGGAACAAC
		muc2.1-R	GTGCTGAGGTTGCAGAATGA
Mucin 5.1 (muc5.1)	XM_009297795.1	muc5.1-F	TGGCAACTTGGCTGATGATA
		muc5.1-R	TCGTCACACGGACCAGTAGA
Mucin 5.2 (muc5.2)	XM_009297793.1	muc5.2-F	GGTGTCTGTTCCGATCAATC
		muc5.2-R	TCATCCTTGTCGCCATTGTA
Mucin 5.3 (muc5.3)	ENSDARG00000089847	muc5.3-F	GGGGAAAACTACACCAGCAA
		muc5.3-R	TGTGAATTCTGTGCCAGAGC
Lysozyme-C (lyz-c)	AF402599	lyz c-F	AAGCAGGTTTAAGACCCACCGAGT
		lyz c-R	AAGTCTGAACAGGCCACTTTGCAC
Heat shock protein (hsp70)	AB062116.1	hsp70-F	CATGGTCCTGGTGAAGATGAA
		hsp70-R	GTCTGTGGGACTCGTTGAAATA
Heat shock protein 90, alpha (cytosolic), class A member 1, tandem duplicate 1 (hsp90aa1.1)	NM_131328.1	hsp90aa1.1-F	CATCGCTAAATCTGGCACAAAG
		hsp90aa1.1-R	GCCACCAGATACGCAGAATAA
Heat shock protein 90, alpha (cytosolic), class B member 1 (hsp90ab1)	NM_131310.3	hsp90ab1-F	GAAGAGGAGAAGGCAGAGAAAG
		hsp90ab1-R	CGAGCCGACATCTTCAATCT
Catalase (cat)	NM_130912.2	cat-F	CCAAGGTCTGGTCCCATAAAG
		cat-R	GCTCAACCTCCGCGAAATA
Super oxide dismutase (sod1)	NM_131294.1	sod1-F	AGGTGACTGGTGAAATTACTGG
		sod1-R	GTCTCACACTATCGGTTGGC
β actin	AF025305	β actin- F	AATCTTGCGGTATCCACGAGACCA
		β actin- R	TCTCCTTCTGCATCCTGTCAGCAA
**Mouse genes**			
Toll-like receptor 2 (Tlr2)	NM_011905	Tlr2-F	CACTATCCGGAGGTTGCATATC
		Tlr2-R	GGAAGACCTTGCTGTTCTCTAC
Toll-like receptor 4 (Tlr4)	NM_021297.3	Tlr4-F	GCTTACACCACCTCTCAAACT

		Tlr4-R	ACAGCCACCAGATTCTCTAAAC
Toll-like receptor 5 (Tlr5)	NM_016928.3	Tlr5-F	GAAGACTGCGATGAAGAGGAAG
		Tlr5-R	CAAGGGTGATGACGAGGAATAG
Myeloid differentiation primary response gene 88 (Myd88)	NM_010851.3	Myd88-F	TCGATGCCTTTATCTGCTACTG
		Myd88-R	GGTCGGACACACACAACTTA
Interleukin 6, transcript variant 1 (Il6)	NM_031168.2	Il6-F	CTTCCATCCAGTTGCCTTCT
		Il6-R	CTCCGACTTGTGAAGTGGTATAG
Interleukin 10 (Il10)	NM_010548.2	Il10-F	TTGAATTCCCTGGGTGAGAAG
		Il10-R	TCCACTGCCTTGCTCTTATTT
Tumor necrosis factor (Tnf), transcript variant 1	NM_013693.3	Tnf-F	TTGTCTACTCCCAGGTTCTCT
		Tnf-R	GAGGTTGACTTTCTCCTGGTATG
Chemokine (C-C motif) ligand 3 (Ccl3)	NM_011337.2	Ccl3-F	GAAGATTCCACGCCAATTCATC
		Ccl3-R	GATCTGCCGGTTTCTCTTAGTC
Defensin, alpha, related sequence 2 (Defa-rs2)	NM_007847.1	Defa-rs2-F	GCATGGAATCTGGGTCAAGATAAC
		Defa-rs2-F	AGAAGGAAGAGCAATCAAGGCTAAG
Defensin, alpha, 21(Defa21)	NM_183253.3	Defa21-F	CCAGGGGAAGATGACCAGGCTG
		Defa21-R	TGCAGCGACGATTTCTACAAAGGC
Cathelicidin antimicrobial peptide (Camp)	NM_009921.2	Camp-F	TCCCTAGACACCAATCTCTACC
		Camp-R	GCCACATACAGTCTCCTTCAC
Lysozyme 1 (Lyz1)	NM_013590.4	Lyz1-F	GAAGCACCGACTATGGGATATT
		Lyz1-R	GATCCCACAGGCATTCTTAGAT
Superoxide dismutase 2, mitochondrial (Sod2)	NM_013671.3	Sod2-F	AGCGTGACTTTGGGTCTTT
		Sod2-R	AGCGACCTTGCTCCTTATTG
Catalase (Cat)	NM_009804.2	Cat-F	GATGGTAACTGGGATCTTGTGG
		Cat-R	GTGGGTTTCTCTTCTGGCTATG
Glyceraldehyde-3-phosphate dehydrogenase (Gapdh), transcript variant 1	NM_001289726.1	Gapdh-F	AGGTCATCCCAGAGCTGAACG
		Gapdh-R	CACCCTGTTGCTGTAGCCGTAT
